# Clinical Application of FDM 3D Printing in Surgery and Traumatology: A Systematic Review

**DOI:** 10.1007/s10439-026-04021-z

**Published:** 2026-02-12

**Authors:** Kristián Chrz, Jan Bruthans, Jan Ptáčník

**Affiliations:** 1 1st Surgical Department, General University Hospital, Prague, Czech Republic; 2 Department of Biomedical Technology, Faculty of Biomedical Engineering, Czech Technical University in Prague, nám. Sítná 3105, CZ-272 01 Kladno, Czech Republic; 3 Department of Anesthesiology and Intensive Care, General University Hospital, Prague, Czech Republic

**Keywords:** 3D printing, Additive manufacturing, Surgery, Medical education, Image-guided surgery, Patient-specific models

## Abstract

**Purpose:**

This systematic review aimed to summarize current clinical and experimental applications of Fused Deposition Modeling (FDM) 3D printing in surgery and traumatology.

**Methods:**

Following PRISMA 2020 guidelines, a systematic search was performed in PubMed®, Scopus®, and Web of Science™ for English-language articles published between January 2020 and March 2025. Studies describing the use of FDM printing in surgical or traumatological contexts were included. Data extraction was conducted independently by three reviewers, and inclusion was determined when at least one reviewer supported it.

**Results:**

Out of 1,691 initially identified records, 35 studies met the inclusion criteria. Identified applications clustered into five thematic domains: surgical planning and patient-specific instruments, surgical training and simulation, biomaterials and tissue engineering, device development and prototyping, and comparisons of printing technologies and materials. FDM printing proved effective for preoperative visualization, educational modeling, and rapid prototyping of patient-specific tools, though heterogeneity in methodology and reporting limited direct comparison.

**Conclusion:**

FDM 3D printing represents a versatile and increasingly accessible tool across surgical disciplines. Despite its lower resolution, its cost-effectiveness, ease of use, and expanding range of medical-grade materials make it a practical choice for clinical and educational implementation. Further standardization and validation are essential for broader clinical integration.

## Introduction

In recent years, 3D printing has become a transformative technology with significant potential in surgical fields and medicine in general, such as spine surgery[1], cardiology [2], neurosurgery [3], orthopedics [4], Ear, Nose, and Throat (ENT) surgery [5], maxillofacial surgery [6], colorectal surgery [7], hepatopancreatobiliary surgery [8] etc.

Before starting the printing process, 3D patient data must be obtained, usually from a Computed Tomography (CT) or Magnetic Resonance Imaging scan. These datasets are extracted from the Digital Imaging and Communications in Medicine (DICOM) format. Based on threshold-based segmentation, the relevant output from the imaging modality (e.g., bone window) is converted into Stereolitography (STL) file format [9–11]. The STL is a standard Computer-Aided Design (CAD) format that represents the surface geometry of the model as a triangular mesh and is widely used in 3D printing. It contains only the geometric information of the object’s surface without any color, texture, or material properties, making it suitable for accurate physical reproduction of anatomical structures. This file is then further processed by software specific to the 3D printer.

Additive manufacturing entered clinical practice in the late 1980 s and early 1990 s, when early Stereolithography (SLA) systems were used to produce anatomical models for maxillofacial and orthopedic planning [6, 9]. SLA works by polymerizing a photosensitive resin with an ultraviolet laser, producing highly detailed models. Similarly, Selective Laser Sintering (SLS) uses a high-powered laser to fuse powdered material (e.g., titanium powder) into a strong, solid, often implant-grade object. Fused Deposition Modeling (FDM, also known as Fused Filament Fabrication – FFF) fabricates objects by extruding thermoplastic filament through a heated nozzle, yielding parts that are cost-effective and mechanically robust.

SLA and SLS methods are widely researched in the literature, but their cost, infrastructure requirements, and regulatory complexity limit widespread clinical use [12]. While FDM is characterized by lower resolution and surface detail compared to resin-based methods, it offers inexpensive hardware, broadly available thermoplastics, and relatively simple handling with minimal post-processing. This makes it particularly suitable for in-hospital 3D printing and point-of-care prototyping [13], although it has been less extensively studied to date [14]. Since 2020, incremental refinements in printable materials and overall printing performance—such as improvements in resolution and production efficiency—have further enhanced the practical usability of FDM [15].

Previous reviews have explored additive manufacturing in selected surgical domains or from a materials science perspective. A 2023 systematic review applied the PRISMA 2020 framework but focused exclusively on spinal applications [16]. A 2024 review examined 3D printing in orthopedic trauma surgery but addressed multiple additive manufacturing technologies rather than FDM specifically [17]. Broader engineering-oriented reviews have analyzed FDM processes and materials, but without a clinical or hospital implementation context [18]. To our knowledge, no prior systematic review has focused exclusively on FDM 3D printing in surgical and traumatological practice, applied the PRISMA 2020 methodology to this body of literature, or synthesized evidence across the multiple principal domains considered in our study.

The aim of this systematic review is to provide a structured overview of the current and potential applications of FDM 3D printing in surgical and traumatological fields. This review is primarily intended for clinically active physicians—particularly surgeons and traumatologists—who are considering or already implementing 3D printing in their clinical workflow. It also offers relevant insights for radiologists involved in image segmentation and model preparation. By synthesizing the current evidence on FDM printing, this work aims to guide clinicians in selecting cost-effective and practical approaches to surgical planning, training, and device prototyping, where rapid availability and adequate geometric fidelity are prioritized over maximal surface resolution, which is typically achieved using resin-based printing technologies. This review further aims to inform decision-makers about its feasibility of FDM for in-hospital use.

## Methods

This systematic review was designed and conducted in accordance with the PRISMA 2020 guidelines (Preferred Reporting Items for Systematic Reviews and Meta-Analyses), and the systematic search was performed following the recommendations outlined in the PRISMA checklist [19]. Elsevier Scopus®, Clarivate Web of Science™, and the U.S. National Library of Medicine PubMed® were systematically searched to identify relevant studies on the use of FDM printing in surgery and traumatology. Key terms were combined using Boolean AND and OR operators to refine the search and focus on specific aspects of the research. The keywords included (“FDM” OR “FFF”) AND (“surgery” OR “traumatology”). The search was restricted to peer-reviewed articles published in English between January 1, 2020, and March 31, 2025, to provide a focused overview of recent developments in the research topic. This publication window was defined a priori to reflect contemporary clinical practice and reporting standards, without implying technological superiority across different periods. Inclusion criteria comprised studies investigating the use of FDM 3D printing in surgical specialties and traumatology. Exclusion criteria encompassed studies in veterinary medicine, studies using 3D printing technologies other than FDM, and papers lacking original data or consisting primarily of review articles already addressed in previous literature.

Data collection from the selected studies was performed using systematic text analysis and key information extraction. A standardized data extraction form was developed to record general study details—such as title, authors, publication year, and country of origin. The extraction process focused on the type of 3D printing technology used and its applications in surgical fields and traumatology. Data were organized and analyzed using an MS Excel spreadsheet.

In the first step, titles and abstracts were reviewed to exclude irrelevant studies, followed by a second phase in which full-text analysis was conducted for those meeting the entry criteria. Evaluation was performed independently by each of the authors; a study was included if at least one author approved its inclusion. The selection process was documented using a PRISMA flow diagram [20]. Because most included studies were feasibility or low-level evidence reports, we conducted only a qualitative assessment of bias, focusing on study design, reporting completeness, and reproducibility.

## Results

**Fig. 1:**
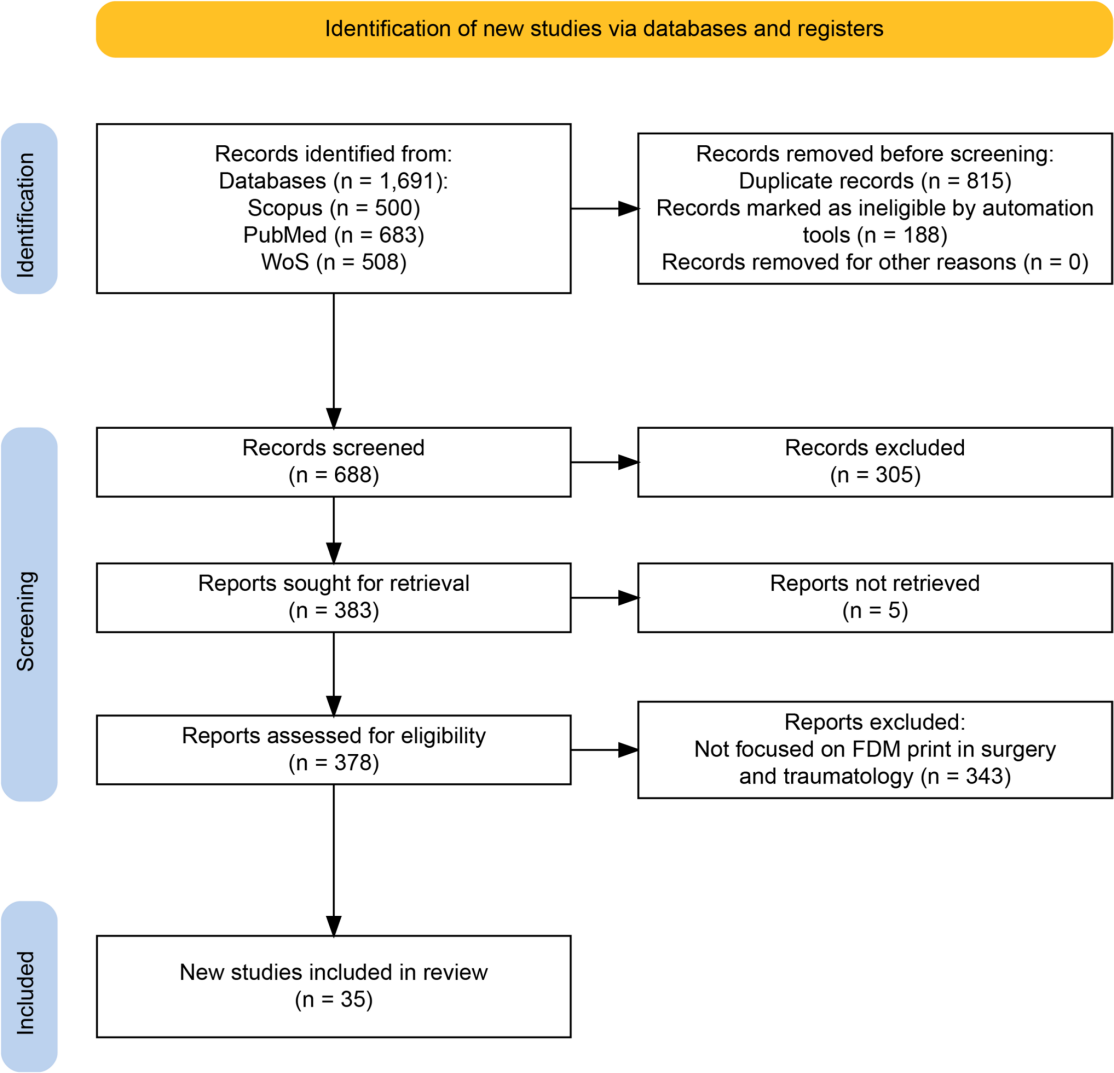
PRISMA 2020 flow diagram

A total of 1,691 sources were identified. After removing 41 non-English sources, 815 duplicates, and 147 sources flagged as inappropriate by automated tools (including letters, interim articles, editorials, corrections, and meeting abstracts), 688 relevant articles remained for further processing. Following title and abstract screening, 305 articles were excluded for not focusing on FDM printing, surgical fields, or traumatology. This left 393 articles for detailed review, 5 of which were inaccessible due to paywalls. The full texts of the remaining 378 publications were systematically analyzed for relevance. Ultimately, 35 articles were included in the study (see Fig. 1). The full list of included studies is presented in Table 1.

**Table 1:** The list of included studies

Author	Article name	Year	Keywords	Summary
S. Weidert et al. [21]	3D printing method for next-day acetabular fracture surgery using a surface filtering pipeline: feasibility and 1-year clinical results	2020	Surgical Planning, Patient-Specific Instruments	Introduced a surface filtering pipeline for 3D printing acetabular fracture models, reducing printing time and cost for preoperative planning
D. A. Salazar et al. [22]	Comparison of 3D printed anatomical model qualities in acetabular fracture representation	2022	Surgical Planning, Patient-Specific Instruments, Comparison of 3D Printing Technologies, Materials	Compared the accuracy and quality of acetabular fracture models from FDM and SLA methods, finding minor accuracy differences but similar information utility for surgical planning
A. Grinčuk et al. [23]	The role of 3-dimensional printed models in the management of the distal radius fractures	2023	Surgical Planning, Patient-Specific Instruments, radius fracture	Compared inter- and intra-observer agreement for distal radius fracture evaluation using 2D/3D CT scans with and without 3D-printed models (FDM, PLA), finding models tend to increase agreement
V.R. Maryada et al. [24]	Pre-operative planning and templating with 3-D printed models for complex primary and revision total hip arthroplasty	2022	Surgical Planning, Patient-Specific Instruments,	Studied the accuracy and utility of FDM 3D-printed anatomical models (ABS plastic) for preoperative planning and simulation in complex primary and revision total hip arthroplasty
R.A. Kovalenko et al. [25]	Comparison of the Accuracy and Safety of Pedicle Screw Placement in Thoracic Spine Between 3D Printed Navigation Templates and Free Hand Technique	2020	Surgical Planning, Patient-Specific Instruments	Compared the safety and accuracy of pedicle screw placement in the thoracic spine using 3D-printed FDM navigation templates (polylactide) versus the free-hand method, finding templates safer
P. E. Eltes et al. [26]	Development of a Computer-Aided Design and Finite Element Analysis Combined Method for Affordable Spine Surgical Navigation With 3D-Printed Customized Template	2021	Surgical Planning, Patient-Specific Instruments, spinal surgery	Proposed a method combining CT, 3D reconstruction, FEA, CAD, and 3D printing to create affordable, patient-specific metal navigational templates for complex spine surgery
K. Y. Lam et al. [27]	Office 3D-printing in paediatric orthopaedics: the orthopaedic surgeon’s guide	2021	Surgical Planning, Patient-Specific Instruments, Pediatric ortopedic	Detailed the setup and workflow for an in-house 3D printing facility in pediatric orthopaedics for surgical planning and education, due to complex pathologies.
L. Frizziero et al. [28]	In-House, Fast FDM Prototyping of a Custom Cutting Guide for a Lower-Risk Pediatric Femoral Osteotomy	2021	Surgical Planning, Patient-Specific Instruments	Proposed an in-house, low-cost FDM 3D printing methodology for custom HTPLA cutting guides in pediatric orthopedic surgery, capable of steam sterilization and reducing risks.
G. C. Menozzi et al. [29]	High-Temperature Polylactic Acid Proves Reliable and Safe for Manufacturing 3D-Printed Patient-Specific Instruments in Pediatric Orthopedics—Results from over 80 Personalized Devices Employed in 47 Surgeries	2024	Surgical Planning, Patient-Specific Instruments,	Documented the in vivo efficacy, usability, and safety of annealed HTPLA 3D-printed patient-specific instruments (3DP-PSI) for pediatric orthopedic surgery, finding them satisfactory and safe
M. O. Subei et al. [30]	A feasibility study of several 3D printing methods for applications in epilepsy surgery	2023	Surgical Planning, Patient-Specific Instruments, Comparison of 3D Printing Technologies, Materials	Compared SLA, FDM, and Polyjet 3D printing methods for patient-specific brain models in epilepsy surgery, assessing efficiency, cost, transparency, and clinical utility
I. R. Belenchón et al. [31]	How to obtain a 3D printed model of renal cell carcinoma (RCC) with venous tumor thrombus extension (VTE) for surgical simulation (phase I NCT03738488)	2020	Surgical Planning, Patient-Specific Instruments	Developed a feasible, accurate, reproducible, and affordable 3D-printed model of RCC with VTE for surgical simulation using FDM technology and flexible PU
N. M. Vitković et al. [32]	The geometrical personalization of human organs 3D models by using the characteristic product features methodology	2024	Surgical Planning, Patient-Specific Instruments,	Integrated CPF and MAF methodologies to create precise, anatomically accurate, personalized 3D geometrical models of human organs (ACL), prototyped via FDM 3D printing
C.R. T. Guzmán et al. [33]	3D printed model for flexible ureteroscopy training, a low-cost option for surgical training	2021	Surgical Training, Simulation Models	Developed a low-cost, 3D-printed plastic model for flexible ureteroscopy training to acquire surgical skills and confidence
R. C. Elisei et al. [34]	A 3D-Printed, High-Fidelity Pelvis Training Model: Cookbook Instructions and First Experience	2024	Surgical Training, Simulation Models	Created and evaluated a 3D-printed anatomical pelvi-trainer from CT scans for basic and advanced minimally invasive pelvic organ surgery training
R. Burgade et al. [35]	Use of 3D printing as a simulation tool for trauma surgery of the pelvis	2021	Surgical Training, Simulation Models	Created a practical educational simulation model of a complex pelvic fracture from CT scans using FDM 3D printing to improve 3D anatomical understanding and surgical training
M. Kiesel et al. [36]	Introducing a novel model for simulating large loop excision of the transformation zone (LLETZ) using 3D printing technique		Surgical Training and Simulation Models	Presented a novel 3D-printed model for realistically simulating diagnostic and surgical interventions (LLETZ) of the cervix to improve gynecologist proficiency
D. Hong et al. [37]	Development of patient specific, realistic, and reusable video assisted thoracoscopic surgery simulator using 3D printing technology	2021	Surgical Training, Simulation Models	Developed a patient-specific, realistic, and reusable 3D-printed phantom for video-assisted thoracoscopic surgery (VATS) training based on CT images
R. B. Inga et al. [38]	Innovator procedure using 3D Design and Printing of bone tissues for educational and hospital applications	2021	Surgical Training, Simulation Models	Created a procedure for manufacturing real-scale FDM 3D-printed bone anatomical models (femur) using minimal resources and software for educational/hospital use
W. Guo et al. [39]	3D printing of polylactic acid-boron nitride bone scaffolds – Mechanical properties, biomineralization ability and cell responses	2023	Biomaterials, Tissue Engineering	Investigated the mechanical, biomineralization, and cell response properties of 3D-printed PLA/Boron Nitride bone scaffolds for tissue engineering
W. Guo et al. [40]	Magnesium Hydroxide as a Versatile Nanofiller for 3D-Printed PLA Bone Scaffolds	2024	Biomaterials, Tissue Engineering	Investigated magnesium hydroxide as a nanofiller for 3D-printed PLA bone scaffolds, evaluating their degradation, biological, and mechanical properties
J. S. Lee et al. [41]	Hybrid 3D bioprinting for advanced tissue-engineered trachea: merging fused deposition modeling (FDM) and top–down digital light processing (DLP)	2025	Biomaterials, Tissue Engineering	Introduced a hybrid 3D bioprinting method combining FDM (PCL) and DLP (hydrogel) for artificial trachea fabrication, demonstrating improved properties and tissue regeneration
I. Rajzer et al. [42]	Biomaterials in the Reconstruction of Nasal Septum Perforation	2020	Biomaterials, Tissue Engineering	Developed a PLLA biomaterial implant using FDM 3D printing for personalized reconstruction of nasal septal perforations, focusing on microstructure and pore size
W. Peng et al. [43]	4D Printed Shape Memory Anastomosis Ring with Controllable Shape Transformation and Degradation	2023	Biomaterials, Tissue Engineering	Developed 4D-printed shape memory anastomosis rings from degradable PLA and PLGA polymers, with tunable degradation and shape memory for optimized anastomotic surgery
J. Kang et al. [44]	Functional design and biomechanical evaluation of 3D printing PEEK flexible implant for chest wall reconstruction	2022	Device Development and Prototyping	Developed a 3D-printed PEEK flexible implant for chest wall reconstruction to restore deformation capability during breathing, evaluating its functional design and biomechanical properties
I. Mayoral et al. [45]	Tissue engineered in-vitro vascular patch fabrication using hybrid 3D printing and electrospinning	2022	Comparison of 3D Printing Technologies, Materials	Developed a hybrid methodology combining 3D printing (FDM) and electrospinning to fabricate patient-specific tissue-engineered in-vitro vascular patches for cell differentiation and functional analysis
B. Flora et al. [46]	Patient-specific cranioplasty, by direct and indirect additive manufacturing of biopolymers and implantable materials	2023	Biomaterials, Tissue Engineering	Evaluated direct and indirect 3D printing methods (PLA, silicone/TPU molds for PMMA) for patient-specific cranioplasty, characterizing surface roughness and mechanical properties
S. S.Y. Pang et al. [47]	Patient-specific 3D-printed helmet for post-craniectomy defect – a case report	2022	Device Development, Prototyping	Case report on the design and use of a patient-specific 3D-printed helmet for post-craniectomy defect, aiding complex surgical recovery
A. Di-Luciano et al. [48]	Trocar shortening for pediatric vitreoretinal surgery with a 3D printed trocar spacer: Report of two cases77	2022	Device Development and Prototyping	Presented results of a 3D-printed trocar spacer (FDM) used for shortening trocars in pediatric vitreoretinal surgery, demonstrating effective shortening in two cases7879
J. I. Rodríguez-García et al. [49]	Transanal endoscopic surgery with a 3D printed device	2021	Device Development, Prototyping	Investigated the effectiveness of ABS 3D-printed devices (FDM) for transanal endoscopic resections without pneumorectum, demonstrating feasibility and safety in a clinical series
L. Tzounis et al. [50]	Three-Dimensional Printed Polylactic Acid (PLA) Surgical Retractors	2020	Device Development, Prototyping	Focused on the preparation and antimicrobial activity of 3D-printed PLA surgical retractors with immobilized silver nanoparticles, developed using FDM technology
L. Frizziero et al. [51]	Heat Sterilization Effects on Polymeric, FDM-Optimized Orthopedic Cutting Guide for Surgical Procedures	2021	Surgical Planning, Patient-Specific Instruments, surgical guides, Comparison of 3D Printing Technologies, Materials	Assessed FDM-optimized orthopedic cutting guides (HTPLA, PLA, nylon) for heat sterilization resistance, finding HTPLA and PLA suitable without compromising geometry
M. Keller et al. [52]	In-hospital professional production of patient-specific 3D-printed devices for hand and wrist rehabilitation	2020	Device Development, Prototyping	Proposed professional mass production of patient-specific 3D-printed devices for hand and wrist rehabilitation, highlighting feasibility and advantages in clinical studies
A. McMillan et al. [53]	Comparison of Materials Used for 3D-Printing Temporal Bone Models to Simulate Surgical Dissection	2020	Comparison of 3D Printing Technologies, Materials	Evaluated different materials (PC, ABS, resins) and methods (FDM, photopolymerization) for 3D printing temporal bone models, assessing anatomical and functional performance for surgical simulation
R. Cafino et al. [54]	Evaluation of polyethylene terephthalate glycol (PETG), Simubone ™, and photopolymer resin as 3D-printed temporal bone models for surgical simulation	2024	Surgical Training, Simulation Models, Comparison of 3D Printing Technologies, Materials	Characterized and compared low-cost 3D-printed temporal bone models (PETG, Simubone™, photopolymer resin) for surgical simulation, finding resin and PETG suitable for certain mastoidectomy procedures
L. Xiao et al. [55]	Polycarbonate–Acrylonitrile Butadiene Styrene Three Dimensional Printing Material Exhibits Biocompatibility and Enhances Osteogenesis and Gingival Tissue Formation with Human Cells	2025	Comparison of 3D Printing Technologies, Materials	Comparing surgical-grade resins with various thermoplasctic filaments (FDM). Identifying specific type of ABS promising material for dental surgery

For clarity, the studies were grouped into five thematic domains reflecting major applications of FDM 3D printing in surgery: **(A)** surgical planning and patient-specific instruments, **(B)** surgical training and simulation models, **(C)** biomaterials and tissue engineering, **(D)** device development and prototyping, and **(E)** comparison of printing technologies and materials. Nevertheless, the boundaries between categories are not always distinct, as several studies overlap multiple domains.

### Surgical Planning and Patient-Specific Instruments (PSIs)

Surgical planning and patient-specific instruments address the need for improved visualization of complex anatomy and more precise intraoperative execution. These applications aim to overcome the limitations of traditional 2D imaging and manual plate bending, particularly in cases of high-complexity fractures or deformities.

Multiple studies have demonstrated the critical role of 3D-printed models in **enhancing preoperative planning**, **increasing surgical accuracy**, and **improving clinical outcomes**. This application spans various surgical specialties, offering enhanced visualization and a high degree of customization.

In **acetabular fracture surgery**, 3D printing aids in visualizing complex fracture patterns and allows for the **preoperative bending of reconstruction plates**, which has been shown to significantly **reduce operation time and blood loss**, as Weidert et al. [21] demonstrated by a method using a surface filtering pipeline with **3D Slicer and FDM printers utilizing Polylactic Acid (PLA)** material. This reduced printing time by 65% —making it cost-effective for routine hospital use. Salazar et al. [22] further investigated acetabular fracture models, proving that even low-cost methods (such as FDM printing) provided sufficient information for surgical planning and were not associated with clinically significant differences in patient outcomes. For **distal radius fractures**, 3D-printed models have been shown to improve **inter- and intra-observer agreement** in evaluating fracture morphology and classification, and are positively perceived by surgeons for preoperative planning and patient communication [23]. Similarly, models used in **total hip arthroplasty** (THA) are highly valued by surgeons as surgical references and for teaching complex procedures [24] .

In **spinal surgery**, FDM-printed navigation templates have demonstrated superior **safety and accuracy for pedicle screw placement** in the thoracic spine compared to free-hand techniques [25]. Using a method that combines CAD and Finite Element Analysis (FEA), individualized metal navigation templates are created for challenging screw insertions [26]. **Pediatric orthopaedics** particularly benefits from patient-specific instruments (PSIs) due to the complexity of pathologies and the uniqueness of pediatric anatomy [27]. L. Frizziero et al. [28] developed a low-cost, in-house FDM 3D printing methodology for pediatric femoral osteotomy cutting guides using **high-temperature PLA** (HTPLA), which can withstand steam heat sterilization. The use of **annealed HTPLA** (ann-HTPLA) has proven to be a reliable and safe material for printing of PSIs in pediatric orthopedic surgery, without significantly increasing infection rates or other complications [29].

For **epilepsy surgery**, 3D-printed patient-specific brain models are valuable for visualizing complex intracranial anatomy and the spatial relationship of implanted electrodes or other surgical tools. These models **assist in post-implantation decision-making** and have been shown to improve communication both among clinicians and between clinicians and patient as the physical models provide a clear, intuitive representation of electrode placement and surgical targets that supports interdisciplinary discussion and patient education [30].

3D models are also valuable for planning complex **renal cell carcinoma (RCC) surgeries with venous tumor thrombus extension (VTE)**, offering a feasible, accurate, and affordable tool for surgical simulation and detailed anatomical visualization [31]. Vitković et al. [32] introduced methodologies for creating **personalized geometrical models of human organs**, such as the anterior cruciate ligament (ACL), using FDM printing to adapt models to specific patient anatomies.

Across these studies, FDM printing consistently enabled clearer anatomical understanding, improved preoperative preparation, and—in several reports—reduced operative time, blood loss, and implant misalignment compared to conventional planning methods.

### Surgical Training and Simulation Models

In surgical education, where cadaver-based training is limited by availability, cost, and logistical constraints, there is a growing need for low-cost, reproducible, and anatomically realistic alternatives. FDM-printed models offer a practical solution, providing accessible, risk-free platforms for skill development that help shorten learning curves and improve procedural confidence.

For **flexible ureteroscopy training**, a 3D-printed plastic model derived from a patient's CT scan allows trainees to practice inserting ureteroscopes, exploring renal and manipulating nitinol baskets for stone removal. One example costs less than €10 and takes 19 hours to print [33].

In **laparoscopic surgery**, an anatomical pelvi-trainer printed using FDM technology provides a life-size, narrow pelvic space environment for both basic and advanced skills training [34]. The model received positive feedback for its utility in urology and gynecology.

**Trauma surgery of the pelvis** can also benefit from 3D-printed simulation models. A 1:2 scale model of a hemi-pelvic fracture, based on CT scans and incorporating bone elements, pelvic vessels, and liquid latex to simulate skin and muscles, provided a realistic 3D representation of the anatomical region. This model enabled the practice of various surgical approaches and plate fixation techniques, with a recommendation for 1:1 scale models for future student training [35].

Other simulation applications include the **Large Loop Excision of the Transformation Zone (LLETZ)** procedure, where 3D printing is used to create realistic models for simulating both diagnostic and surgical steps, aiming to improve training quality for novice surgeons [36]. Similarly, realistic **thyroid cancer and kidney phantoms** have been developed to train residents and assist in surgical planning [37]. **FDM-printed bone anatomical models** [38] are also widely used for educational purposes.

Most studies reported improved trainee confidence and procedural familiarity, with FDM models providing a cost-effective alternative to commercial or cadaver-based simulators.

### Biomaterials and Tissue Engineering

**Tissue-engineering** applications target the limitations of traditional grafts and implants—such as donor-site morbidity and the inability to customize scaffold architecture or degradation behavior—by enabling the fabrication of scaffolds and implants with complex geometries and tailored material properties.

PLA is a widely used material in **bone scaffold applications**. By incorporating boron nitride (BN) nanosheets, composite scaffolds with improved mechanical properties, biomineralization ability, and cellular responses have been developed [39]. Additionally, the use of magnesium hydroxide (Mg(OH)₂) as a nanofiller in PLA bone scaffolds contributes to a weakly alkaline microenvironment conducive to cell growth [40].

For **tracheal reconstruction**, hybrid 3D bioprinting techniques combine FDM for mechanical support (e.g., polycaprolactone [PCL]) with DLP for cell-laden hydrogels (e.g., silk fibroin), resulting in greater flexibility and improved resistance to mechanical forces compared to PCL alone [41].

3D printing also extends to **nasal septum reconstruction**, where poly-L-lactic acid (PLLA) scaffolds can be customized with specific microstructures, layer arrangements, and pore sizes to deliver therapeutic substances or provide structural fixation [42]. This approach addresses the need for alternatives to autografts, particularly in cases of large defects. However, it should be noted that PLLA is a highly crystalline, stereoregular polymer with a higher melting temperature and slower biodegradation rate than PLA, and it requires tighter control of extrusion temperature and cooling than standard FDM printers can provide.

The development of **4D-printed shape memory anastomosis rings** using PLA and Poly(lactic-co-glycolic acid) (PLGA) blends represents an innovative approach to sutureless intestinal anastomosis. These rings offer controllable shape transformation and degradation properties, aiming to reduce complications such as inflammation and stricture associated with conventional techniques [43].

A 3D-printed polyether ether ketone (PEEK) flexible implant has been developed to **mimic costal cartilage** for chest wall reconstruction. Due to its natural-like stiffness, reliable strength, and ability to restore respiratory function, it offers a safer and more functional alternative to rigid implants [44].

Combining FDM with electrospinning (for fine fiber meshes) highlights the integration of complementary technologies to achieve specific material properties required for **vascular patch fabrication** [45].

For patient-specific cranioplasty, 3D printing enables various strategies, including the **direct printing of PLA skullcaps** or the use of **3D-printed molds** (silicone or TPU) for casting poly(methyl methacrylate) (PMMA) prostheses [46]. While all approaches achieve good geometric accuracy, the selected material and manufacturing process significantly influence surface roughness and mechanical properties.

Although largely preclinical, these approaches show potential functional advantages over conventional materials by enabling controlled porosity, drug delivery, or shape-memory behavior tailored to specific clinical scenarios.

### Device Development and Prototyping

**Device development and adaptation of existing medical devices** benefit from rapid prototyping, especially in settings where conventional manufacturing is slow, expensive, or insufficiently customizable for niche surgical needs.

This includes the creation of **patient-specific helmets for conditions such as plagiocephaly, brachycephaly, and post-craniosynostosis**, utilizing 3D scanning or low-dose CT data to ensure a customized fit and support diverse clinical applications [47].

**FDM 3D-printed trocar spacers** for pediatric vitreoretinal surgery provide a faster and potentially safer alternative to time-consuming intraoperative modifications [48].

A customized 3D-printed **device for transanal endoscopic surgery** eliminates the need for pneumorectum, thereby avoiding risks associated with CO₂ insufflation. Although the early-stage study was conducted on only six patients, the device demonstrates potential for cost-effectiveness and customizability [49].

Sterile surgical equipment for various applications, such as **surgical retractors**, can be manufactured from PLA thermoplastic filament with sonochemically deposited silver nanoparticles (AgNPs), offering robust antimicrobial properties against common bacteria along with an on-demand production solution [50].

The development of **custom orthopedic cutting guides** has been a significant area of focus. Research on the effects of heat sterilization on FDM-optimized guides made from polymers such as PLA and HTPLA showed that both PLA and annealed HTPLA can withstand standard autoclave cycles (up to ~ 135 °C) while maintaining geometric integrity [51].

Devices for **hand and wrist rehabilitation** are becoming increasingly feasible through advances in 3D FDM printing technologies [52].

These prototypes demonstrate feasibility and substantial cost reductions compared with traditional machining, though most remain at early proof-of-concept stages with limited clinical validation.

### Comparison of 3D Printing Technologies and Materials

**Comparing printing technologies** is essential for determining whether the lower cost and accessibility of FDM justify trade-offs in resolution, material properties, or anatomical fidelity.

In epilepsy surgery, a **comparison of low-force SLA**, **FDM, and PolyJet Stratasys** printing revealed varying degrees of resolution, transparency, and cost [30]. PolyJet was the most detailed and transparent but also the most expensive; FDM was inexpensive but limited visibility due to its opacity; and SLA was economical and transparent but restricted to single-material printing.

For acetabular fracture models, a **comparison of various printers and segmentation software** (proprietary vs. open-source) showed that high-end resin printers provided superior accuracy over FDM printers in representing fracture fragments, although the differences were often not clinically significant [22].

Studies on temporal bone models for surgical simulation have also **compared different materials and FDM, SLA, and DLP fabrication methods**. Polycarbonate (PC), photopolymerizable polymer (Photo), and white resin (FLW) were found to most accurately replicate surgical drilling characteristics and anatomical detail compared to acrylonitrile butadiene styrene (ABS) and blue resin [53]. Photopolymer resin produced via DLP generally outperformed PETG and Simubone™ (FDM) in terms of anatomical accuracy and dissection experience [54].

**The choice of polymer (PLA, HTPLA, or nylon) and post-processing (e.g., annealing) significantly affect performance during sterilization** as demonstrated on orthopedic cutting guides. Annealed HTPLA demonstrated optimal thermal stability and geometric retention after repeated heat cycles, making it well-suited for reusable tooling, while standard PLA performed adequately for single-use applications due to its lower cost [51].

**Polycarbonate–acrylonitrile butadiene styrene (PC-ABS) demonstrates toughness, heat resistance, surface quality, and low proliferation potential**, making it a promising 3D printing material for dental surgery [55].

Overall, the comparative studies suggest that although resin-based methods offer higher resolution, these differences are often marginal for many clinical planning tasks where FDM provides adequate accuracy at significantly lower cost.

## Discussion

This systematic review identified 1,691 records and, after rigorous screening and eligibility assessment, included 35 studies focusing on FDM 3D printing applications in surgery. The relatively small proportion of eligible publications compared with the initial pool highlights that this is an emerging and still non-standardized field. Despite the increasing accessibility of desktop 3D printers and open-source modeling tools, there remains considerable heterogeneity in reporting quality, study design, and outcome measures. Nevertheless, the included studies collectively demonstrate that FDM technology is increasingly used in routine clinical contexts, showing tangible benefits across surgical planning, training, and device development. Importantly, advances reported since 2020 primarily reflect incremental technical optimisation built upon clinical principles and processes that were already established in earlier work.

Among the most established applications are **surgical planning and patient-specific instruments**, where FDM-printed anatomical models and guides improve preoperative understanding and intraoperative precision. These studies report shorter operating times, reduced blood loss, and enhanced procedural confidence, particularly in orthopaedic, spinal, and craniofacial surgery. Even though FDM lacks the fine resolution of resin-based methods, its affordability and rapid prototyping capability make it particularly suitable for in-hospital manufacturing. The evidence indicates that low-cost FDM workflows, when paired with robust segmentation pipelines, can achieve clinically acceptable accuracy while dramatically lowering production costs.

**Surgical education and simulation** represent another rapidly expanding domain. Training models created by FDM printing enable repetitive, risk-free practice at a fraction of the cost of cadaveric or commercial simulators. While tactile realism remains a limitation—especially for soft-tissue or otologic models—the educational value and accessibility of such tools are consistently emphasized. Their adoption supports the broader shift toward simulation-based surgical training and aligns with competency-based curricula in many specialties.

Emerging **biomaterial and tissue-engineering** studies demonstrate how FDM printing is evolving toward functional, biologically relevant constructs. Thermoplastics such as PLA, highly crystalline PLLA and PCL are now combined with nanoparticles or hydrogels to enhance biocompatibility and mechanical performance, and hybrid systems integrating FDM with electrospinning or bioprinting are appearing. Importantly, these polymers differ in melting temperature, crystallization behavior, and biodegradation profile: standard PLA grades can generally be processed on conventional desktop FDM printers for mid-term scaffolds, whereas PLLA requires a narrower thermal processing window and yields more slowly resorbing, long-term constructs. As a result, not every experimental scaffold reported in the literature is directly transferable to routine, hospital-based FDM workflows. However, most of these works remain preclinical, underscoring the need for standardized evaluation protocols and regulatory pathways before translation into patient care. The same challenge applies to **custom device development**, where prototypes—from cutting guides to implants and surgical retractors—show promising feasibility but limited clinical validation.

Comparative analyses of printing technologies underline a key theme across this review: **FDM printing balances practicality with performance**. Although resin-based printers achieve higher resolution and transparency, the clinical differences are often minor relative to their cost and logistical demands. Consequently, FDM remains the most accessible platform for routine use, especially when sterilization, material availability, and turnaround time are prioritized.

**Dimensional accuracy requirements** in clinical practice are highly application-dependent and are rarely defined using standardized tolerance thresholds. Accordingly, most studies assess FDM accuracy in relation to clinical usability rather than absolute geometric conformity. Reported dimensional deviations for FDM-printed anatomical models typically fall within the sub-millimetric to millimetric range [56], which the reviewed clinical studies indicate is sufficient for spatial understanding and preoperative decision-making (Table 2).

**Table 2:** Reported dimensional accuracy ranges of FDM-printed anatomical models and their reported clinical relevance across representative surgical applications

Clinical application	Reported dimensional deviation	Clinical relevance as reported	Key references
Skeletal anatomical models for preoperative planning	up to ~ 2 mm	Sufficient for spatial understanding and fracture visualization	Weidert et al. [21]; Salazar et al. [22]
Complex fracture models (acetabulum, pelvis)	up to ~ 2 mm	No clinically significant impact on surgical decision-making	Weidert et al. [21]; Salazar et al. [22]
Long-bone and joint models	~ 1 mm range	Improved anatomical orientation and procedural confidence	Grinčuk et al. [23]; Maryada et al. [24]
Patient-specific low-load guides (selected applications)	< 1 mm	Suitable where maximal precision is not required	Frizziero et al. [28]; Menozzi et al. [29]

*Reported dimensional deviations represent indicative ranges derived from the cited studies and are not intended as standardized tolerance thresholds.*

Nevertheless, several inherent **technical characteristics of the FDM printing** must be acknowledged when interpreting its clinical applications. The layer-by-layer deposition process results in anisotropic mechanical behavior and reduced interlayer bond strength compared to bulk manufacturing methods, which can influence the durability of guides or prototypes subjected to loading. Surface roughness and stepped wall geometry may require post-processing—such as sanding or solvent smoothing—particularly for parts intended for close tissue contact. Dimensional accuracy is limited by nozzle diameter, thermal contraction, and slice thickness, making very fine features more challenging to reproduce consistently. In addition, manual removal of support structures can introduce variability in both surface finish and geometry, and it adds to overall fabrication time. Recognizing these limitations is essential for setting realistic expectations regarding FDM’s performance in routine surgical workflows.

This review has several **limitations**. Although a comprehensive search strategy was used and study screening was performed independently by all authors, inclusion decisions based on broad relevance criteria may have introduced some selection bias. The heterogeneity of the included studies—many of which were feasibility reports, technical notes, or small case series with incomplete reporting—limited the ability to perform meta-analyses or direct quantitative comparisons. As a result, the overall evidence base is subject to a high risk of bias, including potential publication bias, as negative or unsuccessful applications are seldom reported. Additionally, some relevant studies may have been missed due to restricted access, indexing limitations, or variable terminology. These factors underscore that the present conclusions should be interpreted qualitatively and highlight the need for standardized reporting, prospective clinical studies, and more rigorous evaluation frameworks in future work.

From a broader perspective, FDM has transitioned from early experimental use to a widely adopted and pragmatic tool for in-hospital 3D printing. Low-cost desktop systems and widely available modeling software have enabled radiology, surgical, and engineering teams to establish local workflows for producing patient-specific anatomical models, cutting guides, and training phantoms. Yet, as this review shows, high-quality, surgically oriented evidence still constitutes only a small fraction of the much larger 3D printing literature, with most studies relying on small series, heterogeneous endpoints, and nonstandardized reporting.

Overall, the diversity of applications and methodological inconsistency observed in the reviewed studies reflect a field in rapid development but lacking established standards. Current evidence demonstrates that FDM can reliably support preoperative planning, surgical training, device prototyping, and experimental biomaterial development at a fraction of the cost of other methods. Future work should prioritize multicenter collaborations, standardized reporting of print parameters, and quantitative cost–benefit analyses. As materials and hybrid printing technologies continue to evolve, FDM is likely to remain a central and democratizing force in surgical innovation—bridging the gap between digital design and clinical practice.

## Data Availability

Not applicable
